# Reversed Corneal Fibroblasts Therapy Restores Transparency of Mouse Cornea after Injury

**DOI:** 10.3390/ijms25137053

**Published:** 2024-06-27

**Authors:** Maria A. Surovtseva, Kristina Yu. Krasner, Irina I. Kim, Nikolay V. Surovtsev, Elena V. Chepeleva, Natalia A. Bondarenko, Alexander P. Lykov, Nataliya P. Bgatova, Alina A. Alshevskaya, Alexander N. Trunov, Valery V. Chernykh, Olga V. Poveshchenko

**Affiliations:** 1 Research Institute of Clinical and Experimental Lymphology Branch of Institute of Cytology and Genetics, Siberian Branch of Russian Academy of Sciences, 2 Timakova Str., 630060 Novosibirsk, Russianataliya.bgatova@yandex.ru (N.P.B.);; 2 Novosibirsk Branch of S. Fedorov Eye Microsurgery Federal State Institution, 10 Kolkhidskaya Str., 630096 Novosibirsk, Russia; 3 Institute of Automation and Electrometry, Russian Academy of Sciences, 1 Academician Koptyug St., 630090 Novosibirsk, Russia; 4 Federal State Autonomous Educational Institution of Higher Education I.M. Sechenov First Moscow State Medical University of the Russian Federation, 2, Building 4 Bolshaya Pirogovskaya St., 119048 Moscow, Russia

**Keywords:** keratocytes, reversion, mouse corneal injury, cell therapy, corneal stromal scar

## Abstract

Cell-based therapies using corneal stromal stem cells (CSSC), corneal keratocytes, or a combination of both suppress corneal scarring. The number of quiescent keratocytes in the cornea is small; it is difficult to expand them in vitro in quantities suitable for transplantation. This study examined the therapeutic effect of corneal fibroblasts reversed into keratocytes (rCF) in a mouse model of mechanical corneal injury. The therapeutic effect of rCF was studied in vivo (slit lamp, optical coherence tomography) and ex vivo (transmission electron microscopy and immunofluorescence staining). Injection of rCF into the injured cornea was accompanied by recovery of corneal thickness, improvement of corneal transparency, reduction of type III collagen in the stroma, absence of myofibroblasts, and the improvement in the structural organization of collagen fibers. TEM results showed that 2 months after intrastromal injection of cells, there was a decrease in the fibril density and an increase in the fibril diameter and the average distance between collagen fibrils. The fibrils were well ordered and maintained the short-range order and the number of nearest-neighbor fibrils, although the averaged distance between them increased. Our results demonstrated that the cell therapy of rCF from ReLEx SMILe lenticules promotes the recovery of transparent corneal stroma after injury.

## 1. Introduction

Eye injuries account for approximately 3% of all emergency department visits, and the majority of these are due to corneal injuries [1]. Injuries can be mechanical and caused by thermal, chemical, and radioactive effects. Severe corneal damage is associated with disruption of the organization of the corneal stroma and can lead to corneal opacity, corneal scarring, and the development of corneal blindness. Corneal blindness is one of the main causes of low vision worldwide [2,3]. According to WHO, corneal diseases occupy fourth place in the structure of visual disability. The relevance and social significance of corneal opacities in the central optical zone is due to blindness and disability of the young working population. This is especially true for developing countries [4]. The main treatment method for patients with corneal blindness is transplantation of a donor cornea. However, a severe shortage of donor corneas and post-transplantation complications limit the use of this treatment method [5]. Therefore, the search for therapeutic approaches related to the development of cellular and cell-free products, bioengineered scaffolds and structures, and corneal prostheses is growing all over the world. Cell therapy using human corneal stromal cells may become an alternative approach to treating corneal stromal pathology, solving a number of problems associated with recovering transparency and lost thickness of the cornea and creating a “healthy cellular microenvironment” in damaged tissue. In animal studies, cell therapy has been shown to improve corneal transparency by recovering native stromal architecture and preventing the development of corneal opacities and scarring [6,7,8,9,10].

The cornea is an avascular, transparent, and well-innervated connective tissue structure. It is involved in light transmission and protection of the structures of the anterior chamber of the eye and also provides two-thirds of the total refractive power of the eye. The transparency of the cornea is due to its well-defined morphological structure and tissue homeostasis [11]. A total of 90% of the corneal volume is stroma. The stroma is formed by densely packed collagen fibers. The stromal fibrils are arranged in bundles (fibrils) and plates (lamellae) parallel to the surface of the cornea. The main cellular representatives of the stroma are keratocytes. Keratocytes make up about 3–5% of the volume of the stroma [12]. Keratocytes are mitotically quiescent, immobile cells. They are of mesenchymal origin and originate from neural crest cells. Keratocytes maintain water balance and tissue homeostasis through the synthesis of growth factors, protein molecules, cytokines, neuropeptides, neurotrophins, and metalloproteinase inhibitors [13]. They are also involved in the synthesis, maintenance, and degradation of the extracellular matrix (ECM), providing morphostructural and biochemical permanence and transparency of the corneal tissue. Keratocytes synthesize ECM components, such as collagens I, III, V, VI, and XII and glycosaminoglycans (keratocan, keratan sulfate, decorin, mimecan, and lumican) [14]. Keratocan and lumican are highly expressed in keratocytes [15] and regulate the transparency and hydration balance of the cornea, organizing and maintaining the topography of collagen fibrils [16].

Injuries, infections, burns, surgical interventions, and other pathological processes can lead to disruption of the integrity of the basement membrane and the entry of IL-1α and TGF-β1, produced by the corneal epithelium, into the corneal stroma. These factors cause disruption of stromal organization. One of the reasons for the transition of keratocytes to an activated state is the effect of TGF-β1 on them [17]. The quiescent keratocytes located at the epicenter of the injury undergo apoptosis, forming an acellular zone in the area of the injury [18]. Keratocytes bordering the site of injury change their phenotype, transforming into an activated state and becoming motile corneal fibroblasts. Fibroblasts migrate to the site of injury, proliferate, and surround the wound with a network of cells [19].

When overstimulated by TGF-β1, fibroblasts can differentiate into myofibroblasts, characterized by the presence of α-SMA and an additional increase in stress fibers. Fibroblasts and myofibroblasts trigger the regeneration of the cornea, causing wound contraction through the rapid synthesis of an opaque extracellular matrix [20]. The corneal regeneration may be accompanied by the formation of scar tissue, which leads to corneal opacification. In the absence of TGF-β1 and IL-1, fibroblasts can transform into quiescent keratocytes after repair of corneal injury. It has been shown that the reversion of fibroblasts into keratocytes can occur not only in vivo but also in vitro when cultivated in serum-free keratocyte growth medium (KGM) [21,22].

The purpose of this work was to evaluate the therapeutic potential of fibroblasts reversed into keratocytes to recover the structural organization of the extracellular matrix and corneal transparency in a model of mechanical injury to the cornea.

## 2. Results

### 2.1. Characteristics of Human Corneal Stromal Cells

**Figure 1:**
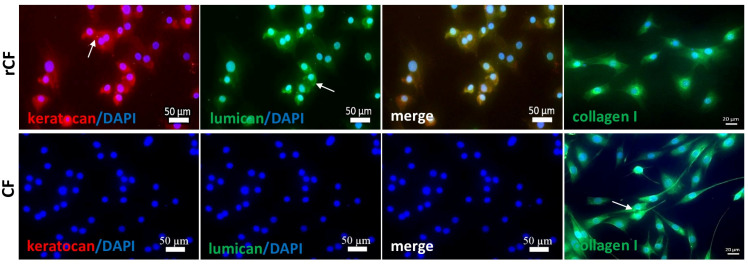
Immunofluorescence microscopy of fibroblasts reversed into keratocytes (rCF) and fibroblasts (CFs). The images show the comparative expression of various markers in cells: red—stained with antibodies to keratocan (arrow); green—staining with antibodies to lumican (arrow) or collagen type I (arrow); blue—DAPI staining of nuclei. The scale bar is 50 µm for keratocan and lumican (the image was captured at 20×) and 20 µm for collagen type (the image was captured at 40×).

Immunocytochemistry confirmed the absence of keratocan and lumican in CF but revealed the presence of type I collagen (Figure 1). Reversed corneal fibroblasts, unlike fibroblasts, highly expressed keratocyte markers such as keratocan and lumican, as well as type I collagen.

### 2.2. Corneal Thickness after Injury and hCSC Injection

**Figure 2:**
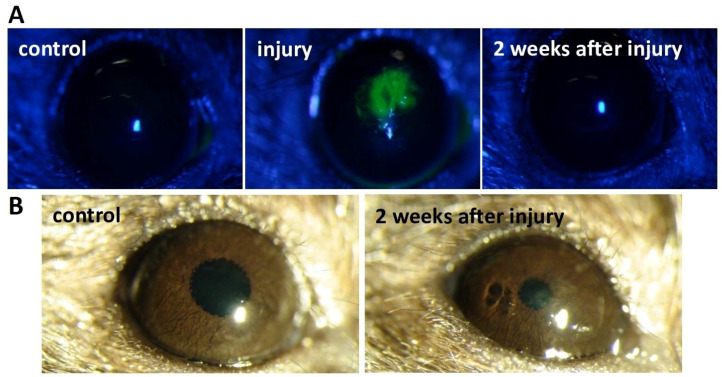
Corneal opacity mouse model. (**A**) Slit-lamp biomicroscopy with fluorescein. (**B**) Slit-lamp biomicroscopy without fluorescein.

The formation of stromal injury in mice (*n* = 32) was confirmed using slit-lamp biomicroscopy with fluorescein (Figure 2A). Lack of fluorescein accumulation was observed in all corneas prior to injury, reflecting the integrity of the epithelial barrier in healthy corneas. Immediately after the injury, a biomicroscopic examination using a slit lamp revealed a local defect of epithelium and stroma, an accumulation of fluorescein dye in the damaged layers, an increased rarefaction of epithelium and stroma, and reactively increasing oedema. We observed complete epithelialization of the wound surface (negative reaction to ocular fluorescein staining) 2 weeks after mechanical injury (Figure 2A). Post-traumatic oedema and rarefaction of the corneal stroma reached their maximum by the second week of observation. Two weeks after the injury, slit-lamp observation demonstrated the development of a haze in the right cornea (Figure 2B). The cornea of the left (control, healthy cornea) eye remained transparent (Figure 2B).

**Figure 3:**
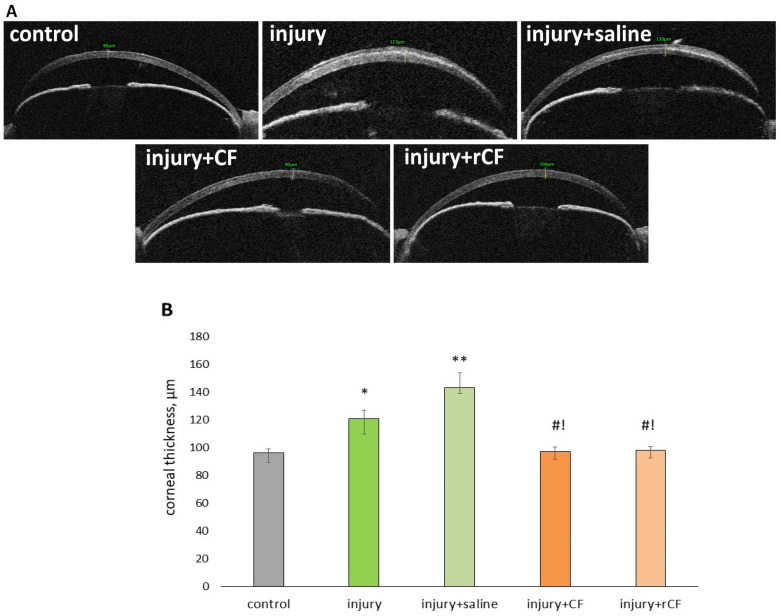
Corneal thickness 2 months after injury and hCSC injection. (**A**) Representative OCT images at months 2 post injury and cell treatment. (**B**) Corneal thickness (µm) at 2 months post injury and cell treatment. Data presented as [Me (Q1; Q3)], (*n* = 8 in each group). * *p* < 0.05 compared to the control (healthy cornea), ** *p* = 0.01 compared to the control (healthy cornea), ^#^ *p* < 0.05 compared to the injury group, ^!^ *p* < 0.05 compared to the injury + saline.

OCT (Figure 3A) showed that the opacities were located in the anterior central corneal stroma of the injured eyes. Corneal thickness (Figure 3B) was significantly higher in the groups with the injury (*p* < 0.05) and injury + saline (sham control) (*p* = 0.01) compared with the control group 2 months after the injury. The corneal thickness in the injury group was [121(115;133)] µm, and in the injury + saline group − [143(132;147)] µm compared to [96(93;103)] µm in the control group. In the groups with the injection of CF and rCF, the corneal thickness was comparable ([97(94;102)] µm and [98(95;104)] µm, respectively) and did not differ from the control.

### 2.3. Corneal Transparency after Injury and hCSC Injection

**Figure 4:**
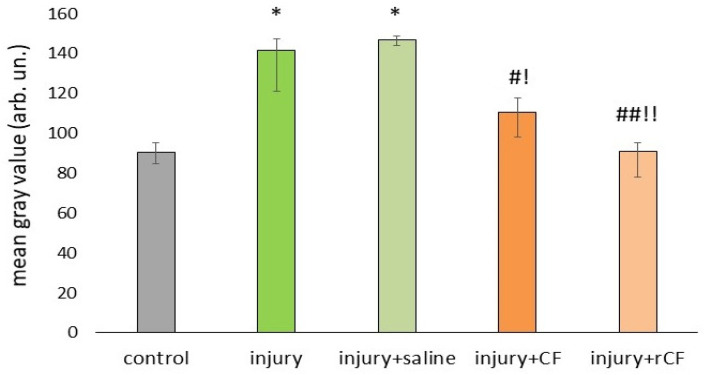
Corneal transparency 2 months after injury and hCSC injection. Mean gray value analysis of OCT data was performed using ImageJ 1.48 v software. Data are presented as [Me (Q1; Q3)], (*n* = 8 in each group). * *p* < 0.001 compared to the control group (healthy cornea), ^#^ *p* < 0.05 compared to the injury group, ^##^ *p* < 0.001 compared to the injury group, ^!^ *p* < 0.05 compared to the injury + saline group, ^!!^ *p* < 0.001 compared to the injury + saline group.

The presence of stromal haze was detected 2 weeks after injury using a slit lamp (Figure 2B). By 2 months of observation, OCT images (Figure 3A) demonstrated high corneal hyperreflectivity in the injury and injury + saline groups in which no injection was performed, a diffuse opaque fibrous component in the corneal stromal layers, and persistent post-injury edema. Corneal hyperreflectivity was weaker in the groups with injection of CF and rCF compared with the injury and injury + saline groups. The hyperreflexivity of the cell injection groups was comparable to the control group. To confirm this, we analyzed the mean gray value of the OCT images using ImageJ 1.48 v software (Figure 4).

OCT examination of corneal opacity 2 months after injury showed a significant increase of corneal opacity in the injury and injury + saline groups compared with control corneas (*p* < 0.001) (Figure 4). Two months after injury and cell injection, the mean gray values in the CF and rCF injection groups were significantly lower than those of the injury and injury + saline groups (*p* < 0.05 and *p* < 0.001, respectively). Moreover, the mean gray value in the cell-injected groups did not differ from the control (healthy cornea). There were also no differences in mean gray value between the CF and rCF injected groups.

### 2.4. Phenotype of Corneas after Injury and hCSC Injection

**Figure 5:**
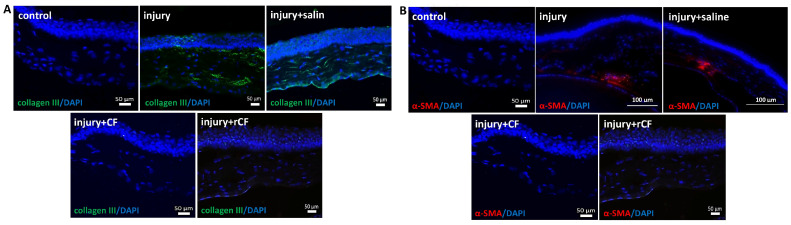
Immunofluorescence analysis of mouse corneal stroma after 2 months of injury and of hCSC injection. (**A**) Immunofluorescence staining of collagen type III in mouse corneal stroma. (**B**) Immunofluorescence staining of α-SMA in mouse corneal stroma. The images show the comparative expression of various markers in cells: green—staining with antibodies to collagen type III; red—stained with antibodies to α-SMA; blue—DAPI staining of nuclei. The scale bar is 50 µm and 100 µm.

To identify markers of fibrosis, we performed immunofluorescence staining of corneal sections for type III collagen and α-SMA two months after injury and cell injection. Immunofluorescence analysis showed stronger staining for type III collagen in the injury and injury + saline groups. Type III collagen and α-SMA were not detected in corneas injected with CF and rCF after injury (Figure 5A). Expression of α-SMA in the injury and injury + saline groups was less pronounced compared to type III collagen expression and was not observed in corneas injected with CF and rCF (Figure 5B)

### 2.5. Transmission Electron Microscopy of Mouse Corneas

**Figure 6:**
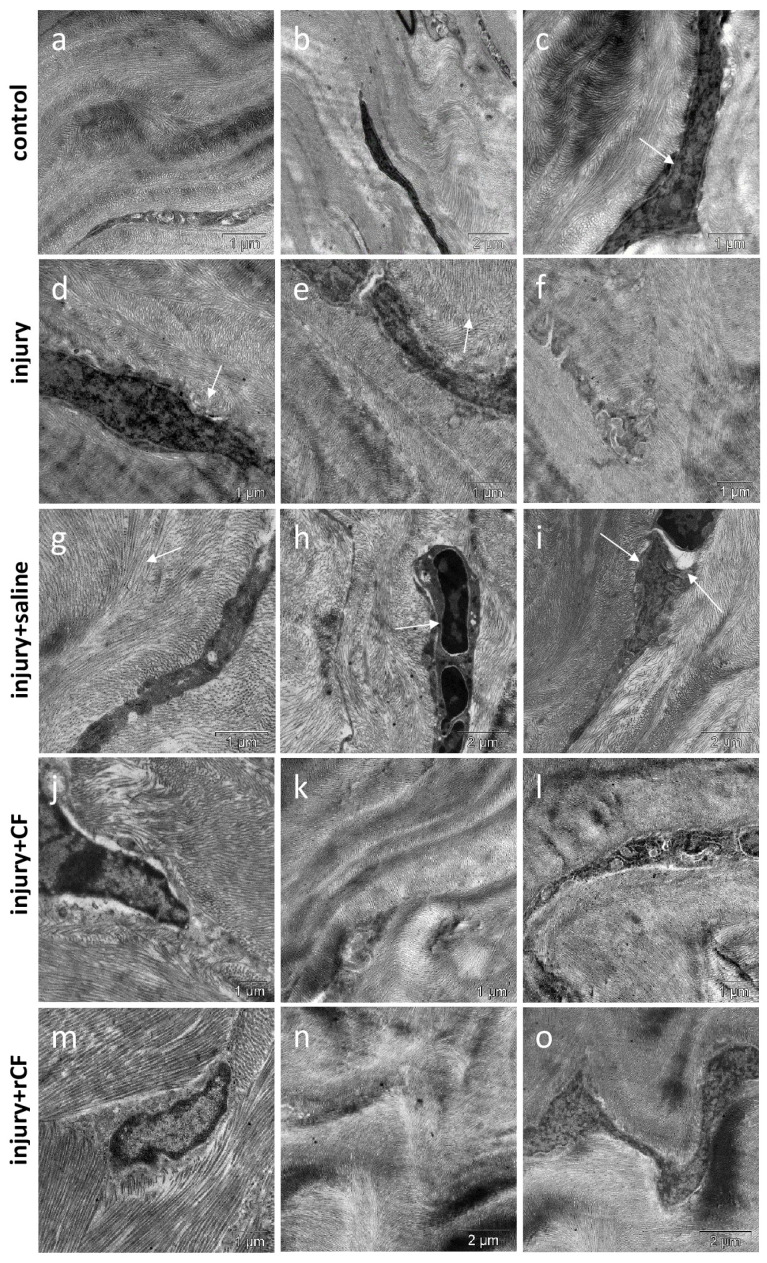
Ultrastructure of keratocytes and extracellular collagen matrix in the mouse cornea 2 months after injury and hCSC injection. (**a**–**c**) “control (healthy cornea)”: (**a**)—ordered arrangement of collagen fibrils around the keratocyte; (**b**)—regular distance between collagen fibrils; (**c**)—keratocyte between collagen fibers (arrow). (**d**–**f**) “injury”: (**d**)—low density and random arrangement of collagen fibrils around the keratocyte (arrow); (**e**)—random arrangement of collagen fibrils (arrow); (**f**)—keratocyte with an increased content of mitochondria in the cytoplasm. (**g**–**i**) “injury + saline”: (**g**)—elongated keratocyte with a disordered arrangement of collagen fibrils and irregular distance between fibrils (arrow); (**h**)—neutrophils in the corneal stroma; (**i**)–keratocyte with swollen mitochondria (arrows), disordered arrangement of collagen fibrils and irregular distance between fibrils. (**j**–**l**) “injury + CF”: (**j**)—low density and random arrangement of collagen fibrils around the keratocyte; (**k**)—structure of the collagen matrix–heterogeneity in the thickness of the collagen fiber bundles; (**l**)—keratocyte with a high content of mitochondria. (**m**–**o**) “injury + rCF”: (**m**)—ordered arrangement of collagen fibrils around the keratocyte; (**n**)—perpendicular arrangement of collagen fiber bundles in the stroma; (**o**)—convoluted form of keratocyte, corresponding to the course of collagen fibers.

The characteristic organization of lamellas was observed in the stroma using transmission electronic microscopy of the control cornea (Figure 6a–c). Collagen fibrils, organized into lamellae, were regularly arranged and had a similar diameter. The interfibrillar distance was constant (Figure 6b).

Keratocytes with a heterogeneous structure were detected in the corneal stroma after the injury. Keratocytes had different shapes and often had a high content of mitochondria, which indicates their active function (Figure 6d,e). The collagen matrix of the cornea was not tightly packed. Collagen fibrils were poorly organized into lamellae and often did not have the regular spacing between fibrils in contrast to native corneal stromal tissue. Collagen fibers showed a disordered growth pattern (Figure 6d–f).

When saline was injected after injury, we observed a reduced density and multidirectional arrangement of collagen fibrils around keratocytes. Keratocytes contained swollen mitochondria with disruption of the cristae structure (Figure 6g,i). Neutrophils were observed in the corneal stroma. (Figure 6h). The collagen fibrils forming the layers of the corneal stroma did not have a well-defined orientation (Figure 6g–i).

Treatment of injury with fibroblasts also results in decreasing the density and multidirectional arrangement of collagen fibrils around keratocytes. Keratocytes contained a large number of mitochondria in the cytoplasm (Figure 6j,l). Heterogeneity in the thickness of the collagen matrix layers was observed (Figure 6k).

When treating injury with keratocytes, the highest degree of maturity of the extracellular matrix of the cornea was observed. An ordered and dense arrangement of collagen fibrils around keratocytes was noted. Stromal fibrils had a unidirectional arrangement and were assembled in layers, and adjacent layers formed an orthogonal lattice (Figure 6m,n). Keratocytes were elongated along collagen fibrils (Figure 6o).

### 2.6. Morphometric Analysis of Transmission Electron Microscopy

#### 2.6.1. Fibril Diameter in the Stroma of Mice

**Figure 7:**
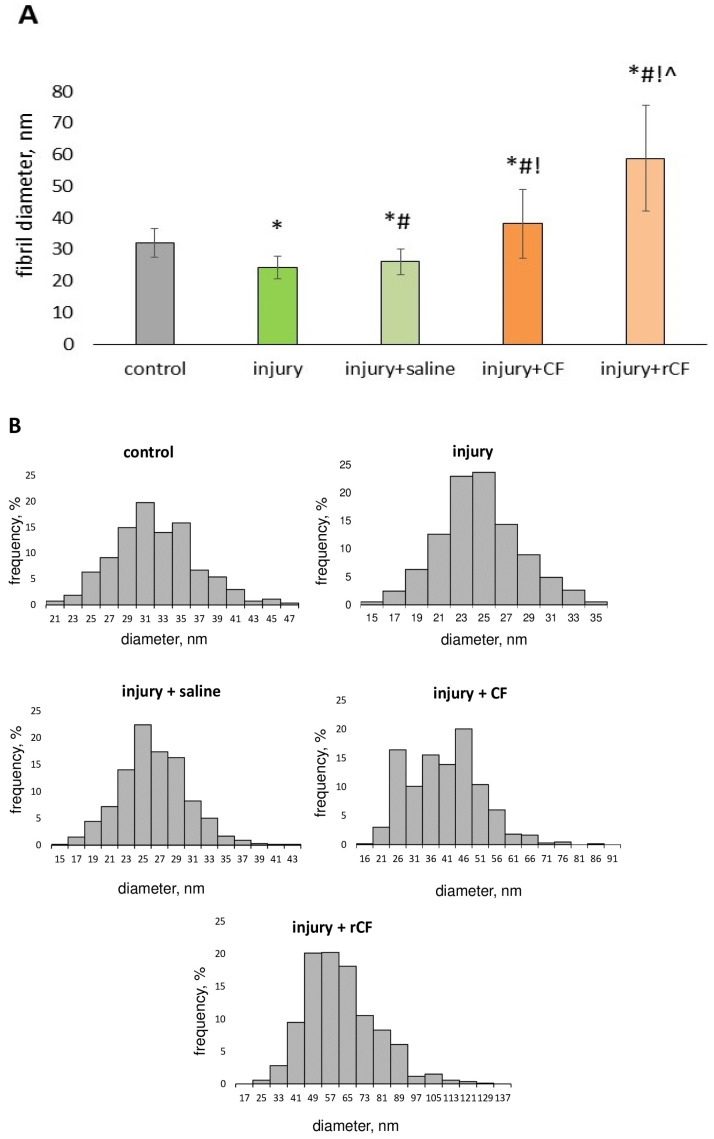
Fibril diameters 2 months after injury and hCSC injection determined with the ImageJ 1.48 v software from TEM images. Five corneas were analyzed with five images per cornea. For each image, at least 100 fibrils were evaluated. (**A**) Data are presented as means ± SDs (*n* > 500 in each group). * *p* = 0.00 compared to the control group (healthy cornea), ^#^ *p* = 0.00 compared to the injury group, ^!^ *p* = 0.00 compared to the injury + saline, ^ *p* = 0.00 compared to the injury + CF. (**B**) Frequency of occurrence of collagen fibril diameter in mouse cornea.

The diameter of collagen fibrils of corneal stroma was analyzed by processing transmission electron microscopy photographs using ImageJ 1.48 v software (Bethesda, MD, USA). A decrease in the diameter of collagen fibrils was found 2 months after injury compared to the control (healthy cornea) (24 ± 3.6 nm versus 32 ± 4.6 nm) (*p* = 0.00). Treatment of corneal injury with cells was accompanied, on the contrary, by an increase in the diameter of the fibrils. The fibrils developed the largest diameter when treated with rCF. The average diameter of fibrils for the injury + rCF group exceeded the diameter of fibrils in the control group by 27 nm (*p* = 0.00), in the injury group by 35 nm (*p* = 0.00), in the injection control (injury + saline) group by 33 nm (*p* = 0.00), and in the injury + CF group by 21 nm, (*p* = 0.00) (Figure 7A).

The distribution of fibril diameter is presented in Figure 7B. Data show that in control corneas, fibril diameters ranged from 21 nm to 47 nm, with 65% of fibrils having a diameter between 29 nm and 35 nm (*n* = 536). In the injury group, fibril diameters ranged from 15 to 35 nm, with 47% of fibrils having a diameter of 23–25 nm (*n* = 570). When saline was injected, 55% of the fibrils had a diameter of 25–29 nm (*n* = 655). During cell injection, a marked increase in fibril diameter was detected. In the injury + CF group, the fibril diameter ranged from 16 nm to 76 nm (*n* = 663), and 50% of the fibrils had a diameter of 36–46 nm. In the injury + rCF group, fibril diameter varied from 19 nm to 123 nm (*n* = 856), with a frequency of 58% for diameters 42–65 nm.

#### 2.6.2. Fibril Density in the Stroma of Mice

**Figure 8:**
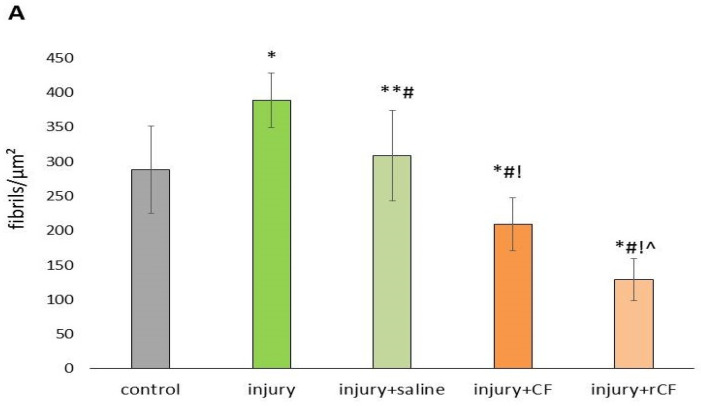

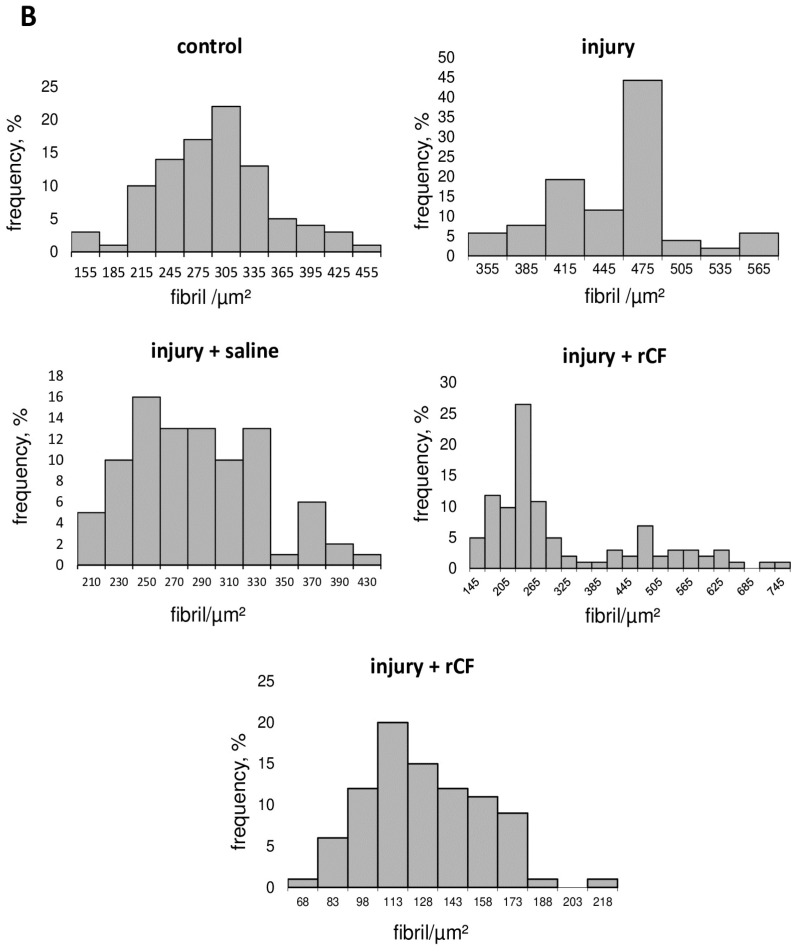
Fibril density 2 months after injury and hCSC injection determined with the ImageJ 1.48 v software from TEM images. Five corneas were analyzed with five images per cornea. For each image, at least 100 fibrils were evaluated. (**A**) Data are presented as means ± SDs (*n* > 500 in each group). * *p* = 0.00 compared to the control (healthy cornea), ** *p* < 0.05 compared to the control (healthy cornea), ^#^ *p* = 0.00 compared to the injury group, ^!^ *p* = 0.00 compared to the injury + saline group, ^ *p* = 0.00 compared to the injury + CF group. (**B**) Frequency of occurrence of collagen fibril density in the mouse cornea.

Two months after the injury, not only was a decrease in the diameter of the fibrils observed but also an increase in their density in the corneal stroma (Figure 8A). Fibril density of the injury group increased compared to the control cornea (388 ± 39.3 versus 288 ± 63.3 fibrils/µm^2^, respectively) (*p* = 0.00). Treatment with cells was accompanied by a decrease in fibril density in the stroma compared to the injury group (*p* = 0.00). It should be noted that treatment with rCF led to a decrease in fibril density by 2.2 times compared to control corneas (129 ± 30.7 versus 288 ± 63.3 fibrils/ µm^2^) (*p* = 0.00) and 1.6 times compared with the injury + CF group (129 ± 30.7 versus 209 ± 38.3) (*p* = 0.00). However, fibril density was significantly lower in the injury + CF group compared with the control and injury + saline groups.

The frequency distribution of fibril density showed that in the control corneas (*n* = 536), 76% of fibrils were packed with a density of 215–335 fibrils/µm^2^, while in the case of stromal injury, 74% of fibrils were packed at a density of 415–475 fibrils/µm^2^ (*n* = 570) (Figure 8B). The number of fibrils in the injury + saline group was comparable to the control group and reduced compared to the injury group. Injection of saline to injury resulted in packing of 77% fibrils with a density of 230–330 fibrils/µm^2^ (*n* = 655).

Treating injury with cells was accompanied by a decrease in the number of fibrils in the stroma (*p* = 0.00). When the injury was treated with CF, a packing density of 175–295 fibrils/µm^2^ was found in 56% of fibrils (*n* = 663), and when treated with rCF, 79% of fibrils were packed with a density of 98–173 fibrils/µm^2^ (*n* = 855) (Figure 8B).

#### 2.6.3. Nearest Interfibrillar Distance in the Stroma of Mice

**Figure 9:**
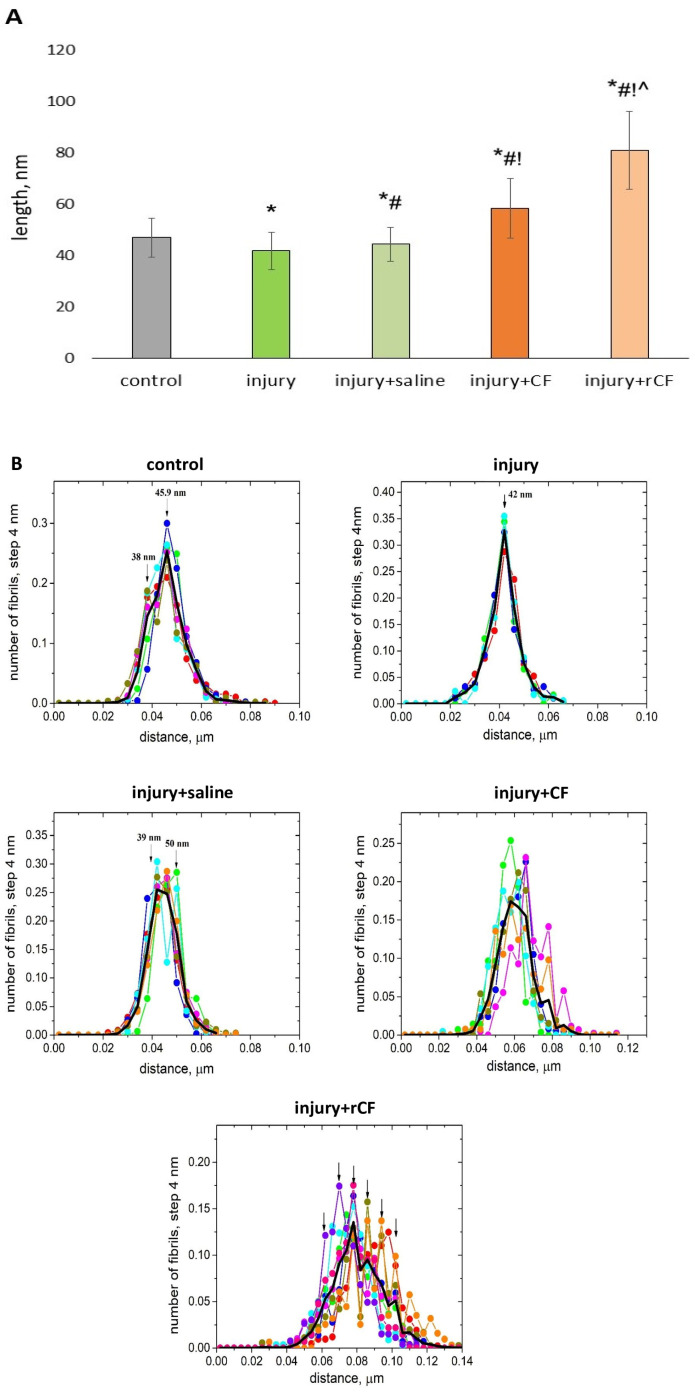

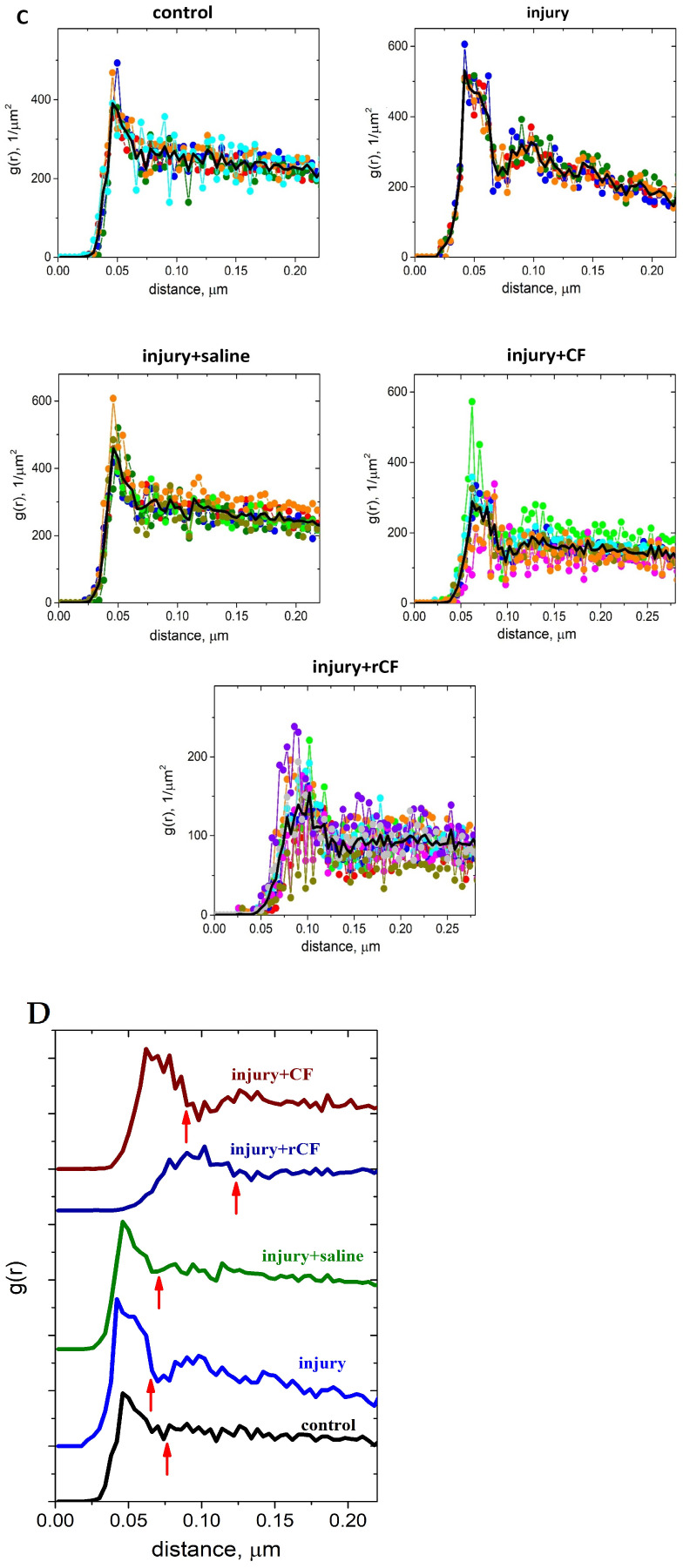
Interfibrillar distance analysis 2 months after injury and hCSC injection determined with the ImageJ 1.48 v software from TEM images. Five corneas in each study group with five or more images per cornea were analyzed. At least 300 fibrils were evaluated for each image. (**A**) Mean distance to the nearest neighbor between fibrils. Data are presented as a means ± SDs (*n* > 500 in each group). * *p* = 0.00 compared to the control group (healthy cornea), ^#^ *p* = 0.00 compared to the injury group, ^!^ *p* = 0.00 compared to the injury + saline group, ^ *p* = 0.00 compared to the injury + CF group. (**B**) Distributions of the nearest neighbor interfibrillar distances. Different curves in each frame correspond to calculations from different photographs. Arrows indicate apparent maxima of the distributions. The thick black lines are the averages of the different curves. (**C**) Fibril pair correlation function *g*(*r*). Each symbolized curve corresponds to data from one photograph. From 300 to 500 fibrils were analyzed in each photograph. (**D**) Averaged pair correlation functions. The arrows show the radii of the circles within which the four closest neighboring fibrils are located.

Mechanical intrastromal injury, as well as the saline injection after injury, were accompanied by a significant decrease in the nearest neighbor distance between fibrils. In the injury group, the mean distance to the nearest neighbor was 42 ± 7.2, and in the injury + saline group, this mean distance was 44 ± 6.5, while the mean distance to the nearest neighbor in the control group was 47 ± 7.7 nm (*p* = 0.00) (Figure 9A). In contrast, treatment with cells resulted in a significant increase in the mean nearest interfibrillar distance. In the injury + rCF group, the average of the nearest neighbor distances was increased by 1.7 times compared to the control group (81 ± 15.1 versus 47 ± 7.7 nm, respectively) (*p* = 0.00) (Figure 9A).

#### 2.6.4. Distribution of the Nearest Neighbor Interfibrillar Distance

The distribution of the nearest neighbor interfibrillar distances, *F*(*R*_0_), was obtained from the micrographs. This distribution represents the probability that, for a given fibril, the distance to the nearest fibril is within a given distance interval with a given distance step. Figure 9B shows the distribution of the nearest neighbor distances for the control cornea. It can be seen that the most likely value is around 45.9 nm, and there are some fibrils having a nearest neighbor at 38 nm. This means a tendency towards a bimodal distribution. In some areas of the fibrils, bimodality is more pronounced, and in others, it is less so (for example, the blue line). In the average distribution (solid black line), the bias towards the bimodal distribution appears as a shoulder towards short distances. The distribution of R0 is asymmetric with a shift toward larger distances, so the average value (47 ± 7.7 nm) exceeds the maximum values. In the case of injury, the shortest interfibrillar distances show a surprisingly narrow unimodal distribution. The data for the four photographs of fibrils agree remarkably well with each other (Figure 9B). In the injury + saline group (Figure 9B), individual fibrils again followed a bimodal distribution, but with the position of the second maximum greater than for the control. In the averaged distribution, the bimodality is not so obvious due to the symmetry of the peaks, but the width of the distribution is significantly wider than for the injury group and closer to the control group (44 ± 6.5 nm).

In the injury + CF group (Figure 9B), multimodality is also evident, although the mean shortest distance is significantly less compared to the injury + rCF group. A feature of this figure is that there is a peculiar region with a narrow distribution and mean value, similar to the groups without cell injection.

Treatment with rCF leads to a strong increase in the nearest neighbor distances with high heterogeneity of *F*(*R*_0_), as seen in Figure 9B. Fibrils tend to form assemblies with a certain distance to the nearest neighbor. Some of the counted regions show strong multimodality. Thus, the distribution of the nearest neighbor interfibrillar distance in the group injected with cells demonstrated wider variation in interfibrillar distance and higher multimodality compared with the injury, injury + saline, and control groups.

#### 2.6.5. Fibril Pair Correlation Function *g*(*r*)

The radial distribution function *J*(*r*) determines the number of fibrils located at a distance r from a given fibril in a layer of thickness dr. In the continuum approximation, which is valid for large distances, *J*(*r*) increases in proportion to the circumference. It is convenient to consider the pair correlation function, *g*(*r*), which excludes this trivial dependence:

*g*(*r*) = *J*(*r*)/2π*r*

At large distances, *g*(*r*) turns into number density (number of fibrils per area). Wide regions in photographs with at least 300 fibrils were selected for *g*(*r*) analysis, and narrow regions were avoided to exclude a border-induced drop in *g*(*r*).

Figure 9C shows *g*(*r*) obtained for the fibril positions of the control cornea. Each curve corresponds to a region of fibrils in a separate photograph. The pair correlation function has a first peak corresponding to the first coordination circle, which is formed by the near-neighbor fibrils. The shoulder near 38 nm and the first maximum at 46 nm in *g*(*r*) correspond reasonably well to the distribution of the nearest neighbor distances for the control in Figure 9B. The first coordination peak ends at approximately 70 nm; at larger distances, *g*(*r*) tends toward a continuum-like behavior. Figure 9C shows that the *g*(*r*) values of different fibers are similar, and the thick black line is the averaged pair correlation function.

The pair correlation function of the injury group shows the bimodal character of the first coordination peak. Peaks that may be associated with the second and third coordination circles are also visible in Figure 9C. The tendency for *g*(*r*) to decrease at large distances may be a consequence of the insufficient number of fibrils participating in the *g*(*r*) estimation (unfortunately, in the photographs of these samples, most of the fibrils were poorly oriented). In the case of the injury + saline samples (Figure 9C), the pair correlation function is qualitatively similar to that of the control sample. In the injury + rCF group, *g*(*r*) is shifted and broadened compared to the control cornea. This corresponds to a significant spread of interfibrillar distances. In the case of the injury + CF group, a similar effect was observed, but to a lesser extent.

The averaged pair correlation functions of different groups are compared in Figure 9D. The curves are vertically shifted for clarity. The curves of the injury + CF and injury + rCF samples are multiplied by a factor of 1.5 for visibility. The arrows in this figure mark the radius of the circle within which the four nearest-neighbor fibrils are located. It can be seen that in all samples, the first coordination circle is formed by the four nearest fibrils. We can conclude that injury and various treatments do not change the coordination number of fibrils, while the interfibrillar distance can vary significantly. From the data presented in Figure 9D, the mean distance for the four nearest-neighbor fibrils can be calculated. We found this distance to be 62.1 nm for the control, 49 nm for injury, 52.7 for injury + saline, 69 nm for injury + CF, and 93 nm for injury + rCF.

## 3. Discussion

With injuries, burns, infections, and degenerative diseases, the cornea loses transparency, and, therefore, visual acuity decreases. Directly or indirectly, this is due to a decrease in the number of stromal cells in the corneal tissue, as well as a violation of their functional properties [23]. Cell therapy is aimed at recovering both the number and functional properties of corneal stromal cells. Previous studies have shown that cell therapy of corneal injuries with hCSSC or hCSC prevented scarring in animal models [6,7,8,9,10]. Cell therapy is a promising direction in the treatment of corneal blindness due to the ability of cells to survive and differentiate into keratocytes and synthesize new collagen and extracellular matrix components in the recipient’s stroma, including the proteoglycans lumican and keratocan [24,25]. For corneal regeneration, the source of cells can be hCSSC [6,7,9], the combination of hCSSC with keratocytes [10], and corneal keratocytes themselves [8]. However, the number of mitotic quiescent keratocytes in the cornea is small, and it is very difficult to increase them in vitro in a quantity sufficient for transplantation. Therefore, we propose to use rCF as a source of cells for cell therapy. We have previously shown that the reversion of CF obtained from ReLEx SMILe lenticules into keratocytes is accompanied by the acquisition of the phenotype and functional properties of keratocytes. hCF reverted to keratocytes when cultured in vitro in serum-free KGM (keratocyte growth medium) [26].

Here, we used rCF to evaluate the therapeutic potential of cell therapy in preventing scarring after injury in a mouse model. In contrast to CF, rCF expressed keratocan and lumican. rCF supported the production of the major component of the ECM in the stromal cornea, collagen I (Figure 1). In our mechanical injury model, we reproduced epithelial-stromal injury to the mouse cornea. First, the epithelial layer was removed in the central zone of the cornea using a 40% glucose solution, and then the stromal injury was modeled with a sharp 33G needle in the form of a tunnel defect in the thickness of the cornea. Damage of the corneal epithelium and Bowman’s layer is accompanied by the release of IL-1, TGF-β1/2, TNF-α, and PDGF, which penetrate into the stroma and lead to remodeling of the stromal extracellular matrix (formation of opacities and scarring) [27,28,29]. These cytokines and growth factors modulate the phenotype, localization, and viability of keratocytes in the corneal stroma. Directly at the wound site, keratocytes undergo apoptosis [30]. Quiescent keratocytes at the wound edges are activated into reparative fibroblasts, which actively proliferate and migrate into the wound [31]. Excess TGFβ1/2 signals from damaged epithelium and apoptotic keratocytes promote the transformation of CFs into αSMA-positive myofibroblasts. CF and myofibroblasts excessively synthesize new proteins, such as fibronectin, α-SMA, tenascin-C, collagen types III and IV, fibrillin-1, biglycan, etc., which cause rapid contraction of the wound matrix and wound closure. However, excessive synthesis of proteins and their disordered deposition are accompanied by a disruption in the organization of fibrils, which leads to opacity of the cornea [32].

In our study, the mouse model of stromal injury mimicked changes similar to those in the human cornea during injury, associated with the formation of opacities and decreased corneal transparency. Slit-lamp observation revealed complete epithelialization of the corneal wound and the presence of stromal haze two weeks after injury (Figure 2). Persistent corneal opacity in the injury and injury + saline groups was observed over a 2-month period on slit-lamp examination. Loss of corneal transparency after injury was also confirmed by OCT (Figure 3A). The corneas of mice that did not receive cell injection after injury (injury and injury + saline groups) were thicker (Figure 3B). In addition, these groups showed high hyperreflectivity of the cornea and higher rates of corneal opacity score (Figure 3A). The mean gray values of the injury and injury + saline groups were significantly higher than those of the cell injection groups (Figure 4). Corneal opacity of mice in groups without cell injection after injury was associated with collagen III synthesis and the presence of myofibroblasts. This is evidenced by immunofluorescence analysis, which showed the presence of collagen III and α-SMA-positive cells in corneal injury, as well as in the injury + saline groups (Figure 5). The thickness and transparency of the cornea in the groups with cell injection did not differ from the corresponding indicators in the control group (healthy cornea) (Figure 3B and Figure 4). In addition, neither collagen III nor α-SMA-positive cells were detected in the corneas of mice from the CF and rCF injection groups by immunofluorescence (Figure 5).

The transparency of the cornea is mainly due to the organization of collagen fibrils in the stroma. Therefore, the next stage of our work was associated with the assessment of the stromal organization of post-injury corneas. When the stroma is recovered after injury, a temporary matrix is assembled, the composition and structure of which differs from the intact stroma. During regeneration, fibroblasts quickly synthesize extracellular matrix, which is deposited in a less organized manner [20]. There is a decrease in the synthesis of keratocan and, in parallel, an increase in the production of decorin and chondroitin. This modified ECM promotes fibroblast migration; however, its altered biochemistry and structure can cause corneal opacity. For tissue remodeling, collagen I production by CF and myofibroblasts is increased [33]. Collagen III expression in CF increases and reaches the highest values in myofibroblasts. Gradually, the temporary matrix is replaced by stromal components, including collagen types I and III, IV, V, VI, etc., to restore the physiological function of the cornea [33,34]. However, uncontrolled proliferation of fibroblasts and myofibroblasts negatively affects stromal function by disrupting the strictly ordered arrangement of fibrils (stromal morphology), thereby altering corneal transparency. In our study, mechanical injury to the cornea was also accompanied by a violation of its morphology. Thus, TEM showed that the injured corneas without cell injection were characterized by a higher density and multidirectional arrangement of collagen fibrils around keratocytes compared to the group with rCF injection and control (Figure 6). Collagen fibrils are poorly organized into lamellae that do not have a clear orientation relative to each other. Keratocytes in groups without cell injection contained a large number of mitochondria, indicating intensive synthesis of ECM components (Figure 6d–i). This may indicate that the process of corneal recovery 2 months after the injury has not yet been completed. Connon et al. showed that central wounds to the full depth (including endothelium) of the rabbit cornea caused by trephine healed over 16 months [35].

The transparency of the cornea also depends on the small diameter of the fibrils, regular interfibrillar distance, and short-range order of collagen fibrils [36,37]. In our study, corneal injury without cell injection was accompanied by a decrease in fibril diameter, an increase in fibril density, and a decrease in the nearest neighbor interfibrillar distance compared to controls (Figure 7, Figure 8 and Figure 9A). At the same time, the distribution of the nearest neighbor interfibrillar distance had a monomodal distribution (Figure 9B). The pair correlation function of the injury and injury + saline groups had a bimodal character of the first coordination peak (Figure 9C). Mechanical injury to the cornea, as well as post-injury injection of saline, did not change the coordination number of fibrils (4) in the first coordination circle, although they reduced the average distance to them by 21% and 15%, respectively (Figure 9D).

Cell injection after injury, on the contrary, was accompanied by an increase in fibril diameter, a decrease in packing density, and an increase in the nearest neighbor interfibrillar distance compared to control and injury corneas without cell injection (Figure 7, Figure 8 and Figure 9). A distinctive feature of groups with cell injection is the presence of a variability in the frequency of fibril diameter and density, as well as a wide, multimodal distribution of the nearest neighbor interfibrillar distance (Figure 7B, Figure 8B and Figure 9B). The pair correlation function in the cell-injected groups was shifted and expanded compared to control corneas, consistent with a significant change in interfibrillar distances. These effects were most pronounced in the injury + rCF group (Figure 9C). One of the reasons for the loss of transparency is also the loss of short-range order of collagen fibrils [38]. The order of the fibrils depends on the interactions of the molecular components between the fibrils. Interfibrillar collagens types VI and XII bind to proteoglycans and fibrillar collagens and play an important role in stabilizing the structure of collagen fibrils [28,39]. However, in our study, the change in the interfibrillar distance was not accompanied by a change in the coordination number of fibrils upon cell injection. The first coordination circle in these groups was formed by the four nearest neighbor fibrils (Figure 9D), although the average distance for the four nearest neighbor fibrils was increased. According to the TEM data, when cells were injected into the injured cornea, the fibrils were arranged in an orderly fashion (Figure 6). This may indicate the synthesis of the main components of the ECM and stromal collagens, which ensure the ordered arrangement of fibrils in the post-injury stroma. Citron et al. showed that healing of the rabbit cornea is accompanied by the appearance of large-diameter fibrils that do not interfere with the preservation of the transparency of the cornea. The process of regeneration of the rabbit corneal stroma was very long and was observed for almost 1.5 years after the injury. Moreover, the variability of fibril diameter during the regeneration of a post-injury cornea is very similar to the variability of fibers in the fetal stroma [40]. It can be assumed that the increase in fibril diameter observed in our study during cell treatment is associated with uncompleted process of stromal regeneration, when increased synthesis of collagen and its packaging into fibrils is still observed.

On the other hand, increased fibril diameter may be due to a deficiency of collagen V. It has been shown that in healing corneal tissue, there is an increase in the content of type V collagen compared to type I collagen [41]. The high content of type V collagen in corneal collagen fibrils is one of the factors responsible for the small and uniform diameter of the fibrils [42], and its decrease leads to the assembly of large diameter fibrils with a wide size distribution [42,43]. It is believed that an increase in the diameter of collagen fibrils and a change in their number can lead to a loss of corneal transparency [36]. The present study shows that neither fibril diameter nor collagen fibril spacing is associated with corneal opacification during wound healing, as previously thought. Our data are consistent with the data obtained in [38]. Our results suggest that although fibril diameter increased, light scattering did not increase sufficiently to cause the cornea to become opacity. It is possible that the unfavorable effect of increasing fibril diameter on transparency was compensated by maintaining cylindrical fibrils and their regular packing. A similar result of maintaining corneal transparency with increasing fibril diameter and regular packing was observed in the Col5a1+/− haploinsufficient mouse model and in patients with classic Ehlers-Danlos syndrome (EDS), associated with a type V collagen mutation [44].

The morphological changes that we identified in the groups with the injection of rCF and CF did not affect the transparency of the cornea. It was previously shown that human cells injected into the stroma of rodents are able to remain alive in the stroma of animals for up to 1–3 months after injection and produce keratocan, lumican, and collagen I [8,45]. We hypothesize that the therapeutic effect (restoration of corneal thickness and transparency) of the injection of rCF into the injured cornea may be associated with the synthesis of extracellular matrix and stroma components by these cells.

The therapeutic effect of CF injection after corneal injury was unexpected for us. We assume that this may be due to their plasticity. In the mouse stroma, in the absence of certain stimuli (e.g., TGFβ), but in the presence of paracrine influence of surrounding mouse keratocytes and microenvironmental factors, human CF become quiescent keratocytes and begin to produce components of the corneal matrix and stroma. We previously showed that CF isolated from ReLEx SMILe lenticules were CD90 (Thy-1) negative cells [26]. CD90 plays a role in wound repair and fibrosis [46]. In many organs, including the eye, the presence of Thy-1–Positive and Thy-1–Negative populations of fibroblasts has been shown [47]. There is evidence in the literature that CD90-negative fibroblasts have less pronounced fibrotic activity. On the one hand, CD90^−^ cells produce 2–3 times less collagen than CD90+ fibroblasts. On the other hand, CD90^−^ cells produce large amounts of fibronectin, which plays a role in recruiting fibroblasts to the site of inflammation. Only CD90+ cells are expressed as α-SMA in the human parental myometrial fibroblasts, orbital fibroblasts [48], and dermal fibroblasts [49]. Long-term expression of CD90 was also observed in pathological scars. It was assumed that CD90+ fibroblasts play an important role in the formation of skin scar in keloids because they preferentially collagenize scars [50].

Thus, injection of CF and rCF to the cornea immediately after mechanical injury protected against scar formation and promoted the recovery of corneal thickness and transparency in mice. Recovery of corneal transparency upon injection of hCSC after injury was associated with the transition of corneal stromal cells from a fibroblastic and myofibroblastic phenotype to a keratocyte phenotype, a decrease in the synthesis of collagen III, and an improvement in the structural organization of collagen fibers in the corneal stroma.

The mechanisms of restoration of corneal transparency after cell injection are unclear and require further study and longer follow-up to better understand the regenerative properties of hCSC. It is also necessary to determine the reasons for the increase in the diameter of collagen fibrils. This may be incomplete regeneration of the corneal stroma or collagen V deficiency. In our study, the cells were administered immediately after injury, whereas in clinical practice, patients with corneal injury do not always receive medical care promptly after injury. Therefore, the therapeutic effect of rCF in the long-term post-injury period, when the formation of opacities is detected, requires further study. The encouraging results obtained in restoring corneal transparency in mice using rCF injection require confirmation of the therapeutic effect in larger animals, such as pigs, whose ocular structure is more similar to the human eye.

## 4. Materials and Methods

### 4.1. Donor Corneas Lenticules

The research was approved by the Local Ethics Committee of the Federal State Institution “Eye Microsurgery named after. S. Fedorov” (No. 1 dated 14 January 2021) and was carried out in accordance with the principles and provisions of the Declaration of Helsinki. Informed consent was obtained from all myopic patients (*n* = 30) enrolled in this study. The research included 13 female and 17 male patients, aged from 18 to 37 years. Corneal lenticules were obtained during the ReLEx SMILE operation, performed using the technology described by W. Sekundo [51]. The cap thickness was 120 μm with an intended diameter of 7.5 mm, and the average diameter of the refractive lens varied from 6.0 to 7.0 mm. A 90-degree lateral circumferential incision of 2.5 mm at the superior temporal position and a cap sidecut angle of 35–40 degrees were performed using the VisuMaxTM FS laser system (Carl Zeiss Meditec, Jena, Germany). A cutting procedure was then performed, and the refractive lenticule was dissected, separated through a side incision, and removed manually. After the ReLEx SMILE procedure, 60 stromal lenticules with thicknesses of 50–175 µm were obtained. The lenticules were placed in a tube with medium and transported at 4 °C to the laboratory.

### 4.2. Isolation and Culture of Human Corneal Stromal Cell for Injection

CF was isolated from lenticules in accordance with the methodology previously described in [26]. Briefly, lenticules from each patient were cut with scissors and treated with 62.5 U/mL collagenase I (Sigma-Aldrich, St. Louis, MO, USA) supplemented with 2% FCS (HyClone, Logan, UT, USA) at 37 °C for 18–20 h. The tissue debris was removed by filtration through a 100 µm cell filter (BD Falcon, Bergen, NJ, USA). Then the cells were washed twice in phosphate-buffered solution (PBS) and plated on a 24-well plate (TPP, Schaffhausen, Switzerland). CF were cultured in DMEM/F12 supplemented with 10% FCS, 1% Gluta-MAX, 5 mM HEPES buffer (Sigma-Aldrich, USA), and 40 µg/mL gentamycin at 37 °C in 5% CO_2._, with medium replacement every 3–4 days. After reaching 80–90% confluence, the attached cells were separated using a 0.25% trypsin/0.02% EDTA solution (Vector, Russia). Cultures from 3 patients, 3–6 passages, were used for the study.

The fibroblasts reversed into the keratocytes culture was obtained from CF with cultivation in KGM (keratocyte growth medium) containing advanced DMEM/F12, 10 ng/mL human basic fibroblast growth factor (bFGF; Sigma-Aldrich, USA), 1 mM L-ascorbate 2-phosphate (Sigma-Aldrich, USA), and ITS (Insulin-Transferrin-Selenium solution, X100; Gibco, Grand Island, NY, USA), 40 µg/mL gentamycin (Dalkhimpharm, Khabarovsk, Russia), 1% Gluta-MAX (Gibco, USA) during 21 days at 37 °C in 5% CO_2_ conditions, with medium replacement every 3–4 days.

### 4.3. Immunocytochemistry

CF and rCF of 3–6th passages were attached to L-polylysine-coated glass slides, fixed in 4% paraformaldehyde, permeabilized with 0.1% Triton X-100 (Bio-Rad, Hercules, CA, USA) for 20 min at room temperature, and then blocked with 1% bovine serum albumin (Sigma-Aldrich, St. Louis, MO, USA) at room temperature for 1 h. Then, they were incubated with PE human anti-keratocan antibody (1:100, cat. no. LAC553Hu41; Cloud-Clone Corp., Katy, TX, USA) and FITC human anti-lumican antibody (1:100, cat. no. LAB496Hu81; Cloud-Clone Corp., Katy, TX, USA), as well as primary rabbit anti-collagen I antibody (1:200, cat. no. AF 0134, Affinity, Wuhan, China), overnight at 4 °C in a humidified chamber, followed by three washes with PBS. Then, the cells were incubated for 1 h in the dark at room temperature with secondary antibodies (goat anti-rabbit IgG conjugated with Alexa Fluor 488, 1:400, cat. no. ab150077; Abcam, Boston, MA, USA). The cell nuclei were stained with 4′,6-diamidino-2-phenylindole (DAPI, Abcam, Cambridge, UK). The cells were photographed using an Axio Observer microscope (Carl Zeiss, Oberkochen, Germany).

### 4.4. Mice, Corneal Stromal Injury, and Intrastromal Cell Injection

The study was conducted in accordance with the ARVO Statement for the Use of Animals in Ophthalmic and Vision Research. The experiment protocol was reviewed and approved by the Local Ethics Committee of RICEL, Novosibirsk, Russia (No. 184 dated 20/10/2023). Female C57BL/6 mice aged 7–8 weeks (*n* = 32) were provided by the vivarium of conventional animals of the Institute of Cytology and Genetics SB RAS (IC&G SB RAS, Novosibirsk, Russia). Mice were anesthetized by intraperitoneal administration of Zoletil-100 (30 mg/kg; Virbac Sante Animale, Cedex Carros, France) and Rometar (0.25 mg/kg; Bioveta, Ivanovice na Hané, Czech Republic) through a 26-gauge needle. The eyes were rinsed with saline solution, and then the central corneal epithelium was removed with a glucose solution (40%; Dalkhimpharm, Khabarovsk, Russia) for one minute. The stromal corneal injury was performed with a sharp 33-gauge needle (SFM Hospital Products GmbH, Berlin, Germany) in the form of a tunnel defect in the thickness of the cornea in the central optical zone. Antibacterial and analgesic drops were instilled into the conjunctival cavity of the experimental eyes of mice before and after injury. The drops consisted of Signicef (0.5%; Sentiss Pharma, Gurugram, India) and Alcaine (0.5%; Alcon, Worth, TX, USA), each administered in a single drop.

The right cornea of each mouse was used for CF or rCF injection or as a sham (injection of the saline), and the left cornea was used as a non-injured control. Cell suspensions (CF or rCF) containing 2.5 × 10^4^ cells in 2 µL saline, or 2 µL saline only (sham), were injected into the mouse cornea stroma with a 10 μL Hamilton microsyringe (Hamilton Company, Reno, NV, USA).

Corneal response (including haze/opacity formation), TEM, and immunohistochemistry were studied 2 months after the injury. A total of 8 corneas were injected with CF, 8 corneas were injected with rCF, 8 corneas were injected with saline only, and 8 injured corneas were left untreated. Ophthalmological examinations of mice were performed under anesthesia with Zoletil-100 and Rometar in doses comparable to those used for the injury. Two months after the injury, the animals were euthanized by continuous exposure to carbon dioxide, and mouse corneas were collected for TEM and immunohistochemistry.

### 4.5. Ophthalmic Examinations of Mouse Cornea

Before stromal injury and after injury, the functional parameters of the corneas (thickness, transparency, and integrity of the barrier epithelial layer) were assessed using instrumental research methods: biomicroscopy of the anterior segment of the eye using a slit lamp Zeiss SL 120 (Carl Zeiss Meditech, Jena, Germany) with a fluorescein test and optical coherence tomography of the anterior segment of the eye using a RTVue XR Avanti computed tomograph (Optovue Inc., Fremont, CA, USA).

- Slit-lamp examination

We examined the anterior segment of the eye of all mice using a slit lamp under direct and side illumination. The binocular slit lamp provides a stereoscopic, magnified, detailed image of the structures of the eye. The fluorescent test served to assess the presence or absence of corneal ulceration. For the fluorescein test, fluorescein strips (Contacare Ophthalmics and Diagnostics, Vadodara, India) were used to stain mouse corneas for examination in a cobalt filter. Corneal opacity was also assessed based on slit-lamp examination before and after injury.

- OCT

Measuring corneal thickness (pachymetry) and determining corneal transparency were performed using RTVue XR Avanti optical computed tomograph (Optovue Inc., Fremont, CA, USA). Corneal transparency was assessed by quantitative analysis corresponding to corneal hyperreflectivity using ImageJ 1.48 v software (Bethesda, MD, USA). For this purpose, the opacity area of 150–200 μm^2^ was taken from the OCT images of each cornea, where the mean gray value was determined from 0 to 250 (250 corresponds to complete opacity).

### 4.6. Corneal Cryosections and Immunofluorescence Staining

After euthanizing the mice, the corneas were removed and frozen in Tissue-Tek OCT medium (Sakura Finetek, Tokyo, Japan) at −22 °C. Ten micrometer thick sections were obtained using a Microm HM-550 cryostat (Thermo Fisher Scientific, Waltham, MA, USA) and mounted on SuperFrost Plus slides (Menzel-Gläser, Thermo Fisher Scientific, Waltham, MA, USA). Corneal cryosections were fixed with 4% paraformaldehyde (Sigma-Aldrich, Burlington, MA, USA), washed with PBS three times, and permeabilized with 0.05% Triton X-100 (Sigma-Aldrich, Burlington, MA, USA) for 10 min. Cryosections were blocked with 1% bovine serum albumin (Sigma-Aldrich, St. Louis, MO, USA) at room temperature for 30 min and incubated with primary antibodies rabbit anti-collagen III antibody (1:100, cat. no. AF5457; Affinity, Wuhan, China) and primary rabbit anti-alpha smooth muscle actin antibody (1:100, cat. no. A17910; ABclonal, Wuhan, China) overnight at 4 °C in a humidified chamber, followed by three washes with PBS. Then, the cryosections were incubated for 1 h in the dark at room temperature with secondary antibodies (goat anti-rabbit IgG conjugated with Alexa Fluor 647, 1:400, cat. no. S0013; Affinity, Wuhan, China and goat anti-rabbit IgG conjugated with Alexa Fluor 488, 1:400, cat. no. ab150077; Abcam, Boston, MA, USA). The cell nuclei were stained with 4′,6-diamidino-2-phenylindole (DAPI, Abcam, Cambridge, UK). The stained samples were analyzed using an Axio Observer microscope (Carl Zeiss, Oberkochen, Germany).

### 4.7. Transmission Electron Microscopy and Morphometry

This experiment used mouse corneas 2 months after mechanical injury. Five corneal samples per group were fixed with a 4% paraformaldehyde in the Hanks medium and were postfixed in a 1% OsO4 solution (Sigma-Aldrich, Saint Louis, MO, USA) with phosphate buffered saline (pH 7.4) for 1 h, dehydrated in ethanol in ascending concentrations, and embedded in Epon ribbon (Thermo Fisher Scientific, Waltham, MA, USA). Semithin 1 μ sections were prepared using the Leica EM UC7 ultramicrotome (Leica Microsystems, Wetzlar, Germany), stained with toluidine blue, and orientated for electron microscopy. Ultrathin sections with thicknesses of 70–100 nm were prepared using the Leica EM UC7 ultramicrotome (Leica Microsystems, Wetzlar, Germany). They were then contrasted with a saturated aqueous solution of uranyl acetate and lead citrate. Digital photographs were taken using a JEM 1400 electron microscope (JEOL, Tokyo, Japan) (Multiple-access Center for Microscopy of Biological Subjects, Institute of Cytology and Genetics, Novosibirsk, Russia).

Morphometric measurements: five or more images per cornea were analyzed. At least 100 fibrils were evaluated for each image. To measure the diameter and density of collagen fibrils, as well as interfibrillar distance, the entire stroma was analyzed, without isolating the anterior or posterior portion of the corneal stroma. Microphotographs were selected randomly based on fibril orientation (fibrils in cross section). Diameters were measured along the minor axis of the cross-sections using the ImageJ 1.48 v software (National Institutes of Health, Bethesda, MD, USA). When calculating the fibril density, an open test system was used, with a test system step of 170 nm or 285 nm. Fibril density was determined as the number of fibrils normalized to 1 µm^2^. The distance to the nearest neighbor from a collagen fibril was determined from micrographs as the distance from it to the nearest fibril.

### 4.8. Statistical Analysis

Statistical analysis was performed using Statistica 10.0 (Stat Soft Inc., Tulsa, OK, USA). The normality of data distribution was confirmed using the Shapiro–Wilk and Kolmogorov–Smirnov tests. To determine differences between the groups, Student’s two- tailed *t*-test or one-way analysis of variance (ANOVA) was employed. The data were presented as means ± standard deviations (SDs). For nonparametric comparisons, the Mann–Whitney U test was used, and results were presented as medians (Me) and interquartile ranges (Q1; Q3). *p*-values < 0.05 were considered statistically significant.

## 5. Conclusions

The present study demonstrated that intrastromal injection of CF and rCF after mechanical corneal trauma resulted in recovery of corneal thickness and transparency in mice.

Injured corneas that received a saline injection or did not receive a cell injection remained significantly opaque and thicker. Intrastromal cell injection suppressed the accumulation of fibrous scar tissue in the stroma and promoted remodeling of the transparent corneal ECM after injury. Although collagen fibrils after cell injection increased in diameter, they were well ordered and maintained short-range order. Our results demonstrate that cell therapy of hCSC from ReLEx SMILe lenticules is a new approach and tool for the prevention of corneal opacities.

## Data Availability

The data that support the findings of this study are available upon request.
